# Supplementary material for: Polyketide synthase gene clusters in the ericoid mycorrhizal fungus Oidiodendron maius: genome-wide prediction, expression profiling, and functional characterization of knockout mutants
Source: Front Plant Sci. 2026 Sep 16;17:1924430. doi: 10.3389/fpls.2026.1924430 (PMC13623903; doi:10.3389/fpls.2026.1924430)
Supplement: Supplementary file 1 [file SupplementaryFile1.zip › Supplementary Materials/Supplementary Folder 1.zip!/Oidma1_AssemblyScaffolds_Repeatmasked/knownclusterblast/cluster47/jgi.p_Oidma1_35040_mibig_hits.html]

| MIBiG Protein | Description | MIBiG Cluster | MiBiG Product | % ID | % Coverage | BLAST Score | E-value |
| --- | --- | --- | --- | --- | --- | --- | --- |
| AAP03116.1 | putative\_carboxyesterase | BGC0000874 | Blasticidin\_biosynthetic\_gene\_cluster | 30 | 83.7088388215 | 131 | 3.2e-30 |
| ABW96543.1 | putative\_type\_B\_carboxylesterase | BGC0000159 | Tautomycin\_biosynthetic\_gene\_cluster | 29 | 43.8474870017 | 96 | 1.5e-19 |
| ABI94378.1 | TMC\_biosynthetic\_enzyme\_L1 | BGC0000157 | Tautomycetin\_biosynthetic\_gene\_cluster | 32 | 38.1282495667 | 95 | 1.9e-19 |
| ABV91296.1 | putative\_type\_B\_carboxylesterase | BGC0000158 | Tautomycetin\_biosynthetic\_gene\_cluster | 32 | 38.1282495667 | 95 | 1.9e-19 |
| ADC96667.1 | carboxyesterase | BGC0001076 | Liposidomycin\_biosynthetic\_gene\_cluster | 31 | 41.4211438475 | 83 | 9.9e-16 |
| ACQ63629.1 | acyltransferase | BGC0000875 | Caprazamycin\_biosynthetic\_gene\_cluster | 27 | 50.6065857886 | 81 | 2.9e-15 |
| BAJ05894.1 | putative\_acyltransferase | BGC0000872 | A-90289\_biosynthetic\_gene\_cluster | 28 | 59.0987868284 | 78 | 3.2e-14 |
